# Elements of Surgical Pathology

**Published:** 1885-09

**Authors:** 


*Elements of Surgical Pathology*. By AUGUSTUS J. PEPPER. Second Edition. Pp. 532. London: CASSELL & CO. 1884.

The call for a second edition of this work within the year is no more than a just expression of its merits. A few chapters have been re-written, and a good deal of additional matter has been introduced. Books are like living things: if they live and thrive, they go on adding to their bulk. If a few more editions of Mr. Pepper's excellent work are called for, we fear it will cease to be "Elements," and become a Manual. We are far from saying that the additions are not improvements, so far as the work is concerned; but, for the purposes of the student already overburdened, we would strongly urge the wisdom of compactness and brevity. We well know the difficulty in commanding this highest virtue of the author—brevity; and we must give Mr. Pepper full credit for having, so far, justly earned the title to this virtue. We hope that in future editions he will not be led to stray from it.

The work bears clearly the impress of a mind thoroughly and practically familiar with surgical pathology. The language carries everywhere the tone of independent and original thought; while the collocation of matter shows extensive acquaintance with the latest work in all departments. The subjects are treated in easily-followed sequences of concatenated reasoning, and not in isolated excerpts borrowed from larger works, as is too often the case in works such as this. The illustrations, where they are original, are somewhat stiff and wooden; otherwise, they are excellent.

